# Micro-costing of genetic and genomic testing in oncology: a systematic review of laboratory resources and costs

**DOI:** 10.1186/s13561-026-00752-w

**Published:** 2026-03-05

**Authors:** Sook Pin Goh, Jue Ern Chan, Siew Chin Ong

**Affiliations:** 1 Discipline of Social and Administrative Pharmacy, School of Pharmaceutical Sciences, Universiti Sains Malaysia, Penang, Malaysia; 2 Big Pharmacy (Perak) Ptd. Ltd., Ipoh, Perak Malaysia

**Keywords:** Micro-costing, cancer, genetic testing, genomic testing, systematic review, oncology, costs

## Abstract

**Background and Objective:**

This review aimed to synthesise evidence from micro-costing studies of laboratory-based genetic and genomic testing in oncology by identifying the resource components, critically examining measurement and valuation methods, determining key cost drivers, and assessing the overall quality and transparency of existing published studies to inform more comparable cost estimation practices.

**Methods:**

Four large electronic databases, PubMed, SCOPUS, Web of Science, and Cumulative Index of Nursing and Allied Health Literature (CINAHL) were searched and restricted to articles published from 2005 to October 2024. The extracted data from the included studies were synthesized and presented in tables covering study characteristics, direct costs (including resource identification, measurement, and valuation), and cost parameters. Cost estimates were adjusted and converted to 2024 US dollar values. The results were narratively synthesized due to the inapplicability of meta-analysis. The quality of the included studies was assessed with the use of a modified version of the Consensus on Health Economic Criteria (CHEC) Checklist.

**Results:**

This review synthesized data from nine eligible studies. All the included studies were from high-income countries. Results demonstrated inconsistent reporting of both the direct and indirect costs. There was also a significant variation in how the researchers identified, measured and valued the costs of labour, equipment, and supplies. The cost of cancer genetic and genomic testing ranged from $76.91 to $11,431.66, with whole exome sequencing (WES) and whole genome sequencing (WGS) being the most expensive options.

**Conclusions:**

Although most studies scored highly on the modified CHEC checklist, it is important to improve in standardization for the future quality of micro-costing research. Specifically, developing uniform terminology, clear guidelines for laboratory workflows, and frameworks for identifying, measuring, and valuing cost components is critical. The establishment of a standardized micro-costing guideline or framework is essential to address these issues. The availability of accurate and high-quality cost data would enable policymakers to make informed decisions regarding the establishment, implementation, and expansion of genetic and genomic testing services across various healthcare settings and countries.

**Trial Registration:**

PROSPERO CRD42024586802.

**Supplementary Information:**

The online version contains supplementary material available at 10.1186/s13561-026-00752-w.

## Key Points

• This review of micro-costing studies on cancer genetic and genomic testing found that reporting of costs is currently inconsistent and the methods used to identify, measure and value specific costs (like labour, equipment, and materials) vary significantly across studies.

• The overall cost of genetic and genomic testing showed a wide range, with advanced tests like WES and WGS being the most expensive options.

• It is critical to standardize the terminology, laboratory workflows, and frameworks used for measuring and reporting all cost components to make future research more useful for decision-makers.

**Supplementary Information:**

The online version contains supplementary material available at 10.1186/s13561-026-00752-w.

## Introduction

Cancer is one of the leading causes of mortality worldwide, accounting for nearly one in six deaths (16.8%) and more than one in five (22.8%) of fatalities attributed to noncommunicable diseases (NCDs) [1]. This poses a significant societal, public health, and economic burden in the 21st century [1]. Global cancer incidence is expected to reach up to 35 million cases by 2050, which urges the need for enhanced preventive and early detection measures [1]. The high morbidity and mortality associated with cancer are mostly driven by risk factors such as lifestyle, environmental exposures, and genetic predisposition [2]. Therefore, prioritizing the management of these key risk factors is important for reducing the future cancer burden, saving millions of lives, and alleviating the profound societal and economic impact of the disease globally [1].

Genetic and genomic variations significantly influence the predisposition to various malignancies, including breast, colorectal, and ovarian cancer [2, 3]. Advancements in precision medicine have shifted cancer diagnostics from single-gene assays toward more comprehensive testing strategies, such as multigene testing or whole genome sequencing (WGS) [4]. It is important to distinguish between genetic and genomic testing which are often used interchangeably but represent distinct concepts. Genetic testing typically targets specific genes or predefined sets of variants associated with hereditary risks or therapeutic implications, such as BReast CAncer gene 1 and 2 (BRCA 1/2) testing or targeted multigene panels. In contrast, genomic testing examines larger portions or the entirety of the deoxyribonucleic acid (DNA) sequence. For example, whole exome sequencing (WES), which focuses on protein-coding regions, and whole genome sequencing (WGS), which captures nearly the entire genomic sequence [5].

Both genetic and genomic tests increasingly rely on next-generation sequencing (NGS), a collective term for high-throughput, massively parallel sequencing technologies that enable the simultaneous sequencing of millions of DNA fragments. This approach underpins contemporary targeted panels, exome-based, and genome-wide assays [4]. NGS-based genetic and genomic testing is now widely used in oncology for germline screening (identifying inherited mutations conferring cancer risk) and somatic profiling (detecting acquired alterations in tumours to inform targeted therapies) [4]. Hence, these applications have profound implications such as guiding personalised treatment, informing risk-reducing interventions, enhancing cancer screening strategies, and facilitating cascade testing for both the affected patients and their close relatives [6, 7].

Despite its clinical potential, the adoption of NGS-based genetic and genomic testing remains constrained by high costs and limited robust economic evaluations, particularly in publicly funded healthcare systems and resource-limited settings [8–10]. To guide policy decisions and ensure the efficient budget al.location of limited healthcare resources, it is crucial to have a pool of robust economic evidence. However, many existing economic evaluations rely on commercial price lists from genetic or genomic testing companies or other publications, which are less favourable for providing true resource estimates to inform decision-making within publicly funded healthcare systems, as these price lists are often profit-driven [11].

The micro-costing approach is recommended as it can meticulously identify and quantify resource consumption which provides a precise evaluation of resource utilization [12]. This approach involves three main stages: identifying all resources required for testing (e.g., workforce, consumables); accurately measuring each resource (such as through time-and-motion studies); and assigning value to each resource used [13]. Although this method is time-consuming, it can more accurately identify the costs of genetic and genomic testing laboratory procedures, especially when the procedures involve high-cost resources. Transparent and detailed cost estimates generated through micro-costing not only strengthen the validity of economic evaluations but also support equitable reimbursement strategies, reduce financial barriers, and improve access to genetic and genomic testing for cancer patients.

Among the most comprehensive forms of genomic testing are WGS and WES which have broader coverage in the detection of genomic variation but are associated with substantial cost burdens due to complex workflows, high-throughput data processing, and specialized labour requirements [5, 14]. Therefore, it is difficult to estimate the true cost of delivering all these tests as many studies lack transparency and consistency in their costing methodologies. A recent systematic review by Gonzalez et al. [15] highlighted a lack of standardization in reporting the sequencing workflows, resource components, and cost inputs, which limits the cross-study comparisons.

Importantly, these methodological challenges are not solely restricted to WES and WGS but are broadly relevant to laboratory-based genetic and genomic testing in oncology. Common findings across studies showed that consumables frequently account for the largest proportion of total costs (often 60–70%), personnel time is mainly driven by the complex bioinformatic analysis and clinical interpretation, and laboratory throughput strongly influences the per-test costs through economies of scale [15, 16]. Hence, the reported cost differences are not solely because of the variations in laboratory prices or national wage structures. Instead, they often arise from inconsistency in methodological choices especially regarding assumptions on batching optimisation, overhead apportionment, shared equipment utilization, and rigorous quality control protocols. Such inconsistencies limit comparability across studies and reduce the reliability of cost inputs used in economic evaluations.

Nevertheless, disease context materially influences cost structures. For example, Schwarze et al. [16] found that rare disease cases incur higher equipment costs due to lower batching efficiency and more samples per case (e.g., trios), whereas oncology testing drives higher consumable, workforce, and bioinformatics costs from deeper sequencing depths, tumour-normal pairing, and complex somatic variant interpretation. Therefore, this study restricted its scope to oncology to enable a more focused and standardized methodological evaluation across the full spectrum of contemporary clinical tests (including targeted panels, multigene assays, and NGS-based approaches), avoiding confounding from these structural differences while still benefiting from transferable methodological lessons.

Previous methodological reviews have addressed micro-costing across healthcare more broadly [17] or examined costing tools for public health interventions [18], but they did not address the specific methodological complexities of the genetic and genomic laboratory. Thus, we applied the costing framework for pharmacogenomic testing developed by Siamoglou et al. [19] to perform a domain-specific synthesis of how direct cost (consumables) and indirect cost (bioinformatics and data storage) components are identified, measured, and valued. By systematically comparing these methodologies, our study provides the empirical evidence base required to justify the urgent need for standardization called for by both the general micro-costing field and the commentary by Gonzalez et al. [20], who highlighted the several challenges in micro-costing genomic testing.

The objective of this study was to critically evaluate articles that applied a micro-costing approach to identify, measure and value the laboratory costs of various genetic and genomic tests across different cancer types. By identifying key cost drivers and methodological gaps and limitations, this review aims to inform recommendations for implementing genetic and genomic screening in oncology especially in resource-limited settings.

## Methods

This systematic review protocol has been registered with International Prospective Register of Systematic Reviews (PROSPERO) with the registration number CRD42024586802. The Preferred Reporting Items for Systematic Reviews and Meta-Analyses (PRISMA) guideline was followed for conducting and reporting in this review [21].

### Eligibility criteria

The eligibility criteria were based on the PICO (Population, Intervention, Comparator, Outcomes) framework to identify all the relevant studies included in this systematic review study. The detailed inclusion and exclusion criteria are summarized in Table 1.

**Table 1:** Inclusion and exclusion criteria

Category	Details	
	Inclusion Criteria	Exclusion Criteria
Population	• Adult and/or paediatric patients undergoing genetic or genomic testing for any type of cancer.	• Non-cancer genetic and/or genomic testing or mixed populations (e.g., rare diseases), even if cancer data is reported separately.
Intervention	• Laboratory-based genetic and/or genomic testing (e.g., NGS, WGS, WES, multigene panels, liquid biopsy, Sanger sequencing, Fluorescence In Situ Hybridization (FISH), and Chromosomal Microarrays (CMA).	• Non-laboratory genetic and/or genomic testing • Studies failing to detail the specific laboratory workflow.
Comparator	• Not Applicable	
Outcomes	• Detailed breakdown of costs related to laboratory procedures and testing processes. The outcome is the analysis of detailed cost components such as labour, reagents, equipment, and administrative costs involved in the genetic or genomic testing workflow. • Reported cost outcomes (e.g. cost per sample; cost per patient)	• Aggregate costs without a detailed breakdown of laboratory-specific components.
Study Designs	• Primary empirical studies applying a standalone micro-costing approach to laboratory-based genetic or genomic testing for cancer.	• Economic evaluations (e.g., cost-effectiveness analyses, budget impact analyses, cost-utility analyses) where micro-costing is not the primary objective or is not reported as a standalone methodological component. • Studies where laboratory costs are estimated as model inputs rather than empirically measured. • Reviews, editorials, commentaries, conference abstracts, and methodological papers without original cost data.
Language	• English language	• Non-English language publications.
Date of Publication	• From January 2005 until October 2024	• Before 2005 or after October 2024
Country	• No restriction	• Not applicable

Four large electronic databases were applied to search including PubMed, SCOPUS, Web of Science, and Cumulative Index of Nursing and Allied Health Literature (CINAHL) (via EBSCO platform). The searches were conducted in November 2024 and were limited to articles published from 2005 up to October 2024. The year 2005 was chosen as the starting year because NGS was first introduced in that year [22]. A manual search of all the cited references and reference lists of included studies and systematic reviews was performed using Google Scholar to identify additional relevant studies. The systematic literature search was conducted using specific keywords as shown in Table S1 of the Additional File 1 such as “cost”, “genetic testing”, “genomic testing” and “cancer” which were combined with Boolean operators to ensure a comprehensive exploration of the literature. For instance, (cost OR economic) AND (genetic testing OR hereditary test) AND (neoplasm OR cancer). These terms were used both as Medical Subject Headings (MeSH) and free-text words to capture relevant studies addressing the outcomes of interest. The search strategy was initially developed for PubMed and translated and applied to other databases afterward. The search strategy for all databases was summarized and presented in the Table S2-5 of the Additional File 1.

Cost estimates were first adjusted for the cost-year using a gross domestic product (GDP) deflator index and followed by conversion to 2024 US dollars using purchasing power parity (PPP) for currency conversion rates [23]. These adjustments were performed with the use of a cost conversion tool developed by the Campbell Collaboration (CC) and the Evidence for Policy and Practice Information and Coordinating Centre (v.1.7, last updated: January 2024) [23].

Due to the significant heterogeneity among studies in terms of cancer types, workflow processes, and resource classifications, a direct comparison of average testing costs was not feasible. Instead, results were synthesized and presented narratively in the results section.

### Selection of studies and data extraction

All the identified articles were first cross-checked, and the duplicates were removed by two reviewers (SP and JE). The remaining articles underwent independent screening by the two reviewers based on titles and abstracts against the eligibility criteria. Eligible articles were then retrieved in full-text format. Both reviewers (SP and JE) independently screened and assessed the full texts of the articles to determine the studies’ eligibility. Any discrepancies or disagreements between the reviewers’ findings were resolved through discussion with a third reviewer (SC).

A data extraction tool was developed and stored in Microsoft Excel format. Extracted information included bibliographic details, country of origin, type of genetic and/or genomic testing, study design (randomized controlled trial or observational study with or without a comparison group), setting, type of cancer, resources measured and valued, source of costs components, discount rate, total costs, currency, year, type of uncertainty analysis, and measured outcomes. All data were subsequently presented and summarized in a table.

### Data synthesis

The selected studies were categorized based on the country of origin (high-income vs. low- and middle-income countries), cancer type, genetic or genomic testing method, and study design. Key information was extracted such as testing workflows, sensitivity analyses, and study perspectives, along with additional considerations such as equipment depreciation rates, overhead costs, instrument maintenance, as well as bioinformatic expenses. All the key information was cross-tabulated to compare methodologies and resource classifications across all included studies.

**Fig. 1:**
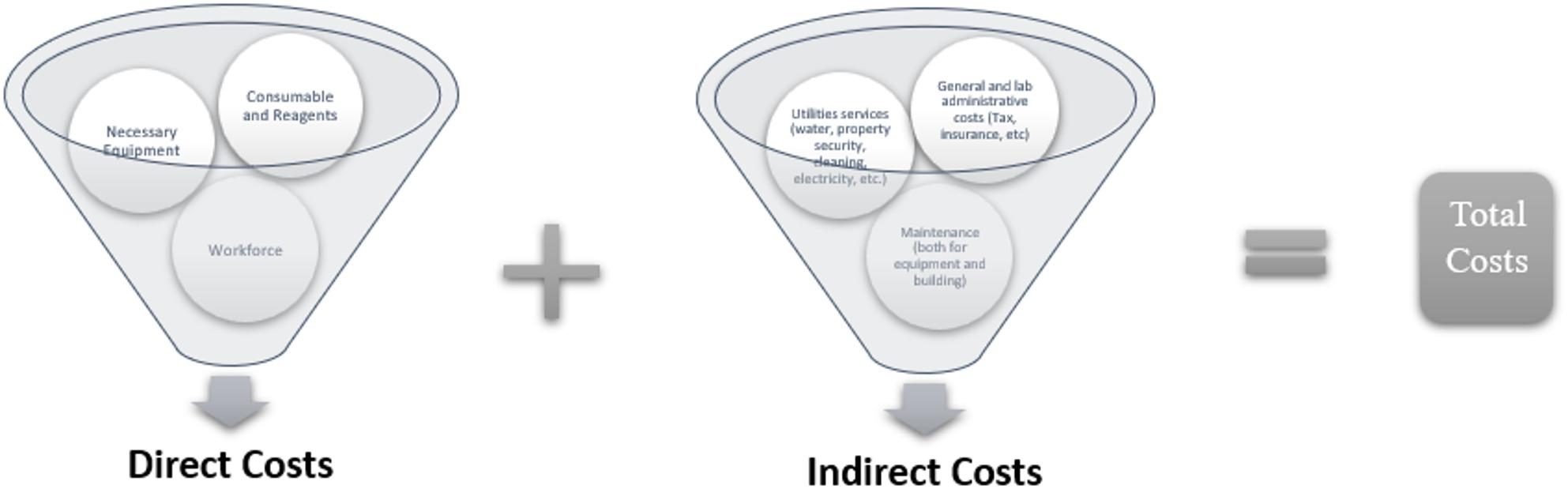
Cost framework for Pharmacogenomic Testing [19]

For each process stage, resource categories were identified, detailing the specific resources included (e.g., consumables/ reagents, workforce, and equipment) and the methods or data sources used for (i) measurement (e.g., interviews, time-and-motion studies) and (ii) valuation (e.g., invoice amounts, hospital financial records, provider price catalogues). Categorization of resources into predefined categories was often difficult due to different studies aggregating and reporting resources in different ways. There was also a lack of consistency in how relevant resources were identified, measured, and valued. The identified resource categories were categorized into direct and indirect costs according to the recommended cost framework for pharmacogenomic testing by Siamoglou et al. [19] as shown in Fig. 1.

### Quality assessment

Currently, there is no consensus or specific quality assessment tool exists for evaluating the methodological quality of micro-costing studies. Therefore, this review employed a modified version of the Consensus on Health Economic Criteria (CHEC) Checklist [24] to assess the quality of the included studies. The CHEC Checklist provides a standard core set of 19 items for evaluating the methodological quality of economic analyses. Given that the included studies focused on micro-costing in the economic evaluation of genetic or genomic testing rather than cost-effectiveness comparisons, items related to the appropriateness of the time horizon (item 5), the quality of outcome assessment (items 10–12), incremental analysis of costs and outcomes with compare (item 13), and the discounting of future costs and outcomes (item 14) were considered not applicable and were therefore excluded [13]. Items 7–9 which assess the extent to which all relevant costs were accurately identified, measured, and valued were expanded to include additional details to enhance the evaluation of cost assessment rigor for all the included studies. This modification was adapted from the framework by Potter S. et al. [13]. While Potter S. et al. [13] used it to evaluate micro-costing for surgical interventions, the present adaptation captured key methodological features specific to laboratory-based genetic and/or genomic testing. These features included the transparency of resource identification, measurement of laboratory and personnel time, valuation of capital and consumables, and reporting of assumptions for the finalised studies.

## Results

**Fig. 2:**
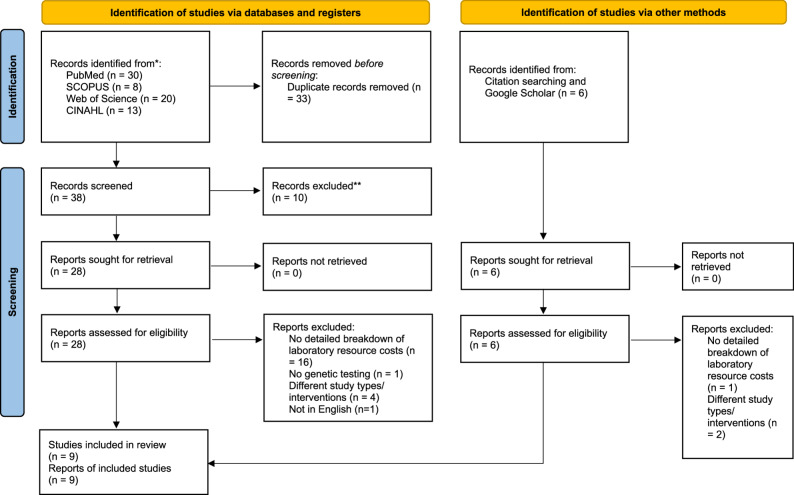
PRISMA 2020 flow diagram screening results

A total of 71 papers were initially identified through database searches. Approximately 38 papers remained after eliminating the duplicates. After title and abstract screening, 28 articles were left for full-text evaluation. In addition, six more studies were found through reference list screening and Google Scholar searches. Consequently, the number of studies eligible for full-text review became 34. Overall, nine studies met the eligibility criteria and were selected for the final analysis. The selection process is illustrated in Fig. 2. Of the 25 studies excluded at full-text review, 17 reported only aggregate cost estimates without detailed laboratory resource breakdowns [25–40], 4 embedded micro-costing within broader economic models without standalone methodological reporting [41–44], 2 evaluated non-genetic screening interventions [45, 46], and 2 included mixed or non-cancer populations [16, 47]. Detailed exclusion rationales are presented in Additional File 1, Table S6.

### Study characteristics

The selected studies were published between 2016 and 2024 [48–56]. All nine studies were conducted in high-income countries [48–56] and more than half originated from Europe [49, 50, 52–54, 56]. The remaining publications were from North America [48, 55] and Oceania [51]. The findings showed that single-gene testing and/or targeted gene panels [49–52, 55, 56] using NGS were the most frequently reported genetic and/or genomic testing methods with up to six articles, followed by WGS [51, 52, 56] in three articles and WES [51, 53] in two articles. In contrast, liquid biopsy [54], chromosome-level analysis [56], and gene expression profiling [48] were less commonly reported, with each method mentioned in only one article.

The selected studies covered a diverse range of cancer types, with several examining various cancers broadly. Three studies [49, 53, 54] did not focus on a specific cancer type. Lung cancer was the most commonly studied, with three [51, 52, 55] out of the nine studies conducting micro-costing analysis on this cancer.

In general, most of the studies applied micro-costing analysis in observational comparative study design [48–52, 55, 56], and only Bayle et al. [53] conducted a case series study. More of the studies collected data retrospectively or prospectively [48–55] and only one study collected data both prospectively and retrospectively [56]. Around five studies collected data from multi-centre [49, 51, 52, 54, 56] settings and the remaining studies collected data from single-centre [48, 50, 53, 55]. Among the selected studies, four studies were conducted in a clinical practice setting [49, 52, 54, 56] and the remaining studies were conducted as research projects [50, 51, 53, 55]. Notably, one study operated in both clinical practice and research [48]. All the included studies could be categorized as studied from a healthcare provider perspective [48–56]. Table 2 summarizes the key information for the study characteristics of the selected micro-costing studies.

**Table 2:** Study characteristics of selected articles

Authors (Year)	Country of Origin	Study Design	Data Collection	Single- centre/ Multi-centre	Type of Genetic or Genomic Testing	Somatic and/or germline testing	Type of Cancers	Setting of cost estimation (clinical practice/research project)	Platform	Perspective	
Costa et al. (2016) [48]	Canada (North America)	Observational comparative study	Prospective	Single-centre	Digital gene expression profiling & fluorescence in situ hybridization​, and targeted capture sequencing	Somatic	Lymphoid Cancer	Clinical practice and Research project	nCounter Digital Analyzer platform (Digital gep); Metafer imaging software & Carl Zeiss Axio Imager Z2 microscope (Dual-colour fish); Illumina MiSeq platform (Targeted capture sequencing)	Provider (BC Cancer Agency)	
Marino et al. (2018) [49]	France (Europe)	Observational comparative study	Prospective	Multi-centre	Targeted gene panels (next-generation sequencing)	Somatic & Germline	Various cancers	Clinical practice	Miseq, PGM, GAIIx, Proton	Healthcare provider	
Ryan et al. (2019) [50]	England (Europe)	Observational comparative study	Prospective	Single-centre	Tumour microsatellite instability testing, immunohistochemistry, and/or MLH1 methylation testing, and germline next-generation sequencing	Somatic & Germline	Lynch syndrome- Endometrial cancer	Research project	Miseq	National Health Service perspective	
Gordon et al. (2020) [51]	Australia (Oceania)	Observational comparative study	Retrospective	Multi-centre	Targeted panels, whole-exome sequencing; whole-genome sequencing	Somatic & Germline	lung, breast, oesophageal cancers, melanoma or mesothelioma.	Research project	Illumina Xten™, NextSeq™, MiSeq™, BGISEQ-500™	Health provider’s perspective (i.e., state government hospital)	
Pasman et al. (2021) [52]	Netherlands (Europe)	Observational comparative study	Retrospective	Multi-centre	Immunohistochemistry, Fluorescence In Situ Hybridization, pyrosequencing, High Resolution Melting, Sanger sequencing, next-generation sequencing gene panels, Cobas, Biocartis, whole-genome sequencing	Somatic & Germline	Non-Small Cell Lung Cancer; melanoma; colorectal cancer; gastrointestinal stromal tumour	Clinical practice	NovaSeq 6000 from Illumina	standard case (An average Dutch laboratory practice)	
Bayle et al. (2021) [53]	France (Europe)	Case series	Retrospective	Single-centre	Whole-exome sequencing	Somatic	Various cancers	Research project	HiSeq 2000 sequencer (2015); NovaSeq 6000 (2018)	Hospital	
Kramer et al. (2022) [54]	Dutch, Netherland (Europe)	methodological framework and case studies	Retrospective	Multi-centre	Circulating tumour DNA (ctDNA)	Somatic	Various cancers	Clinical practice	Polymerase chain reaction-based methods; mass spectrometry-based methods; next-generation sequencing	Healthcare provider	
Kumar et al. (2022) [55]	Canada (North America)	Observational comparative study	Prospective	Single-centre	Targeted gene panels (next-generation sequencing)	Somatic	Non-Small Cell Lung Cancer	Research project	Trusight Tumor 170 Kit, the Oncomine Focus, the QIAseq Targeted DNA Custom Panel, the QIAseq Targeted RNAscan Custom Panel, KAPA HyperPlus/SeqCap EZ (Roche)	Provider	
Thangavelu et al. (2024) [56]	Sweden (Europe)	Observational comparative study	Combination of prospective and retrospective	Multi-centre	Chromosome Banding Analysis, Fluorescence In Situ Hybridization, Targeted Mutation Analysis, Gene Panel Sequencing, Array-Based Copy Number Variation Analysis, Multiplex Ligation-dependent Probe Amplification, whole-genome sequencing	Somatic & Germline	Acute Leukaemia (tumour & germline samples)	Clinical practice	Illumina NovaSeq 6000	clinical	

Next-generation sequencing refers to high-throughput sequencing technologies used for targeted panels, whole-exome sequencing, or whole-genome sequencing; BC Cancer Agency is a provincial cancer agency in British Columbia, Canada.

### Resources classification

All the included studies [48, 49, 51–56] reported both direct and indirect costs, except for Ryan et al. [50], which reported only direct costs (consumables/reagents, equipment, and workforce) in the study.

#### Identification of cost components

There were different identification methods for consumables/reagents and equipment across all the studies. Expert-driven or staff interview methods were the most common method used for consumables/reagents which were reported in four studies [51, 52, 54, 55], and for equipment, reported in three studies [51, 52, 55]. Observation-based methods [48, 49, 53] and data/records-based methods (e.g., hospital databases [50], project records [51]) were also utilized, though less frequently. Notably, several studies did not report how resources were identified, particularly for equipment [53, 54, 56].

#### Measurement methods

Measurement approaches varied substantially across studies. Consumables/reagents and equipment were measured using observed quantities [48, 49, 53], structured data collection tools [50], inventories [50, 51], or estimated quantities [54] informed by expert consensus or interviews [52, 55]. One study did not report measurement methods for consumables/reagents [56], and three studies did not report measurement methods for equipment [53, 54, 56]. Workforce measurement was most commonly based on observation-based methods, including direct observation with standardised questionnaires and time-motion studies [48–50, 55]. Other approaches included staff or project records and public hospital databases [51], activity-based time estimates supported by expert input [54], expert consensus [52], and self-reported workforce surveys [56].

#### Valuation methods

Valuation of consumables/reagents and equipment was frequently based on market prices or supplier list prices (consumables/reagents: three studies [49, 51, 55]; equipment: four studies [49, 51, 53, 55]. Institutional/administrative sources (e.g., financial departments, hospital invoices) were used in two studies for both consumables/reagents and equipment [50, 54] while published or catalogue prices were reported in three studies for consumables/reagents [48, 50, 53] and two studies for equipment [48, 50]. Only one study did not report its valuation methods for both consumable/reagents and equipment [56]. Workforce valuation was primarily based on two methods: standardized wage scales (e.g., mean gross wage [49], annual gross salary [56], common wage scale [51]) or institution-specific financial records & budgets (e.g., an average of the scale of the profession in the collective labour agreement for academic hospitals [54], facility employers’ 2018 costs [52], annual labour budget [53], budget reviews verified by the team [55], and National Health Service (NHS) Agenda for change pay scales, British Medical Association (BMA) Hospital doctor pay scales [50]). Only one study did not report the method used to value workforce costs [48]. Table 3 summarizes the methods of identifying, measuring, and valuing resource categories for direct cost components.

**Table 3:** Direct Costs – resource identification, measurement, and valuation.

	Consumable and Reagents			Necessary Equipment			Workforce		
	Identification	Measurement	Valuation	Identification	Measurement	Valuation	Identification	Measurement	Valuation
Costa et al. (2016) [48]	Direct observation	Quantity recorded during observation	Manufacturer prices	Direct observation	Quantity recorded during observation	Manufacturer prices	Direct observation	Time-motion study	Not stated
Marino et al. (2018) [49]	Direct observation (standardised questionnaire)	Quantity recorded during observation	Purchases prices	Direct observation (standardised questionnaire)	Quantity recorded during observation	Purchases prices	Direct observation (standardised questionnaire)	Time recorded	Mean gross wage
Ryan et al. (2019) [50]	Laboratory SOPs, lab staff input	Structured data collection	Published list prices, hospital invoices	Hospital information systems	Asset and utilisation records	Published list prices, hospital invoices	Direct observation	Time-motion study	NHS agenda for change pay scales, BMA hospital doctor pay scales
Gordon et al. (2020) [51]	Laboratory staff/ project records/ public hospital databases	Resource inventories	Market prices (provided by staff or estimated)	Laboratory staff/ project records/ public hospital databases	Resource inventories	Market prices (provided by staff or estimated)	Laboratory staff/ project records/ public hospital databases	Staff time allocation	Common wage scale
Pasmans et al. (2021) [52]	Expert consensus	Consensus-based quantities	Supplier list prices	Expert consensus	Consensus-based quantities	Supplier list prices	Expert consensus	Estimated staff input	Facility employers’ 2018 costs
Bayle et al. (2021) [53]	Observations	Observed quantities	Catalogue price	Not stated	Not stated	Acquisition costs	Not stated	Not stated	Annual labour budget (mean wage estimate)
Kramer et al. (2022) [54]	Laboratory protocols & expert input	Estimated quantities	Internet/ laboratories and financial departments	Not stated	Not stated	Internet/ laboratories and financial departments	Time measurement of the activity/ expert input	Activity-based time estimates	Average of the scale of the profession in the collective labour agreement for academic hospitals
Kumar et al. (2022) [55]	Provider/staff interviews	Estimated quantities	Market price	Provider/staff interviews	Estimated quantities	Market price	Direct observation, provider/staff interviews	Time-motion study/ reported time	Budget reviews (team-verified)
Thangavelu et al. (2024) [56]	Not stated	Not stated	Not stated	Not stated	Not stated	Not stated	Workforce survey	Self-reported staffing input	Annual gross salary

Abbreviations: *SOPs* standard operating procedures, *BMA* British Medical Association, *NHS* National Health Service.

### Indirect and excluded costs

Among the indirect costs, maintenance costs were the most frequently reported. However, most studies [49, 52–55] considered only the maintenance costs of equipment, excluding building maintenance costs. Kramer et al. [54] assumed a 5% annual maintenance cost, excluding the first year under warranty. Marino et al. [49] calculated hourly maintenance costs using annual contract fees divided by usage hours, while Bayle et al. [53] divided maintenance fees by usage hours to estimate annual costs. Only one study calculated maintenance costs together with equipment costs [55]. Additionally, there was one study that categorized costs related to information technology, security, building operation, and maintenance under overhead costs [48]. Only Kramer et al. [54] included facility (laboratory space) in indirect cost by assuming a 6% markup for housing costs. Utility costs were mostly calculated as a percentage of total costs or based on institutional data. Kramer et al. [54] applied a 38% markup for overhead costs, while Marino et al. [49] used the French hospital cost database to estimate utility costs at 27% of total expenses. Meanwhile, Bayle et al. [53] also reported utility costs as 27% of direct costs derived from the accounting data system. Some studies included additional indirect costs, such as failure rates [54], training [49, 53], research and development [49], software [52], bioinformatics [53, 55, 56], and data management [56].

Only a few studies reported the types of costs excluded [51–54, 56]. The most commonly excluded costs were operational and administrative expenses, such as blood and tumour biopsy collection [52], sample collection [53], DNA extraction from both tumour and blood samples [52], system development [56], implementation and validation [54], overheads [52, 56], management [56], and storage [51, 53, 54]. Other excluded costs included personnel-related expenses [51, 53, 54], infrastructure and equipment costs [51, 56], as well as research and data management costs [51].

### Costs and parameters of genetic and genomic testing

Substantial variability was identified in the costs of genetic and genomic testing across included studies. The unit costs reported varied across studies with costs expressed per case, per person or patient, or per sample, which were depending on type of test, technological application, and costing approach adopted. The overall adjusted cost estimates range for genetic and genomic testing methods ranges from $76.91 to $11,431.66 [48–56]. The cost of circulating tumour DNA (ctDNA) testing was the widest range of cost, varying from $251.44 to $11,431.66 per sample depending on the type of platform, setting, and testing volume [54]. Targeted NGS gene panels varied between $290.97 and $1267.84 per patient [49–51], with per-sample costs ranging from $450.12 to $1290.17 [55], depending on the assay used. WGS costs ranged from $314.31 to $4597.48 per patient [51, 52, 56], with lower costs reported for high-volume analyses ($314.31 per patient for 7500 analyses) [56]. WES costs ranged from $730.35 to $2956.51 per tumour sample [51, 53]. Other tests like Sanger sequencing [52] and immunohistochemistry (IHC) [50] were cheaper and typically under $500 per patient. Digital gene expression profiling and targeted capture sequencing costs varied from $631.85 to $1,089.97 per case [48]. Overall, WES and WGS remain a more expensive approach, while other tests provide a cheaper alternative option.

All the studies that conducted sensitivity analysis used a one-way [50, 51, 55, 56] or deterministic approach [49, 52, 53] except Kramer et al. [54] and Costa et al. [48] who did not specify the type of sensitivity analysis. Regarding cost drivers, all reported studies identified consumables and reagents as key cost drivers [48–53, 55, 56], except for one study that mentioned test volume as the cost driver [54]. In general, the most expensive steps for genetic and genomic testing were library preparation and sequencing [48, 51, 53–56] whereas the least expensive steps reported were blood sample collection [48, 54] and data storage [51, 56]. More than half of the included studies reported the method of equipment depreciation [48, 49, 51–55] except Thangavelu et al. [56] and Ryan et al. [50]. The methods and rates applied for equipment depreciation varied across all the reported studies, with most studies assuming a 5-year lifespan and using linear amortization or straight-line methods, with interest rates ranging from 3% to 4.5% [48, 49, 51–54]. Table 4 and Table 5 summarizes the parameters and cost data of genetic and genomic testing for the included studies.

**Table 4:** Economic parameters and cost drivers

	Costa et al. (2016) [48]	Marino et al. (2018) [49]	Ryan et al. (2019) [50]	Gordon et al. (2020) [51]	Pasmans et al. (2021) [52]	Bayle et al. (2021) [53]	Kramer et al. (2022) [54]	Kumar et al. (2022) [55]	Thangavelu et al. (2024) [56]
Type of Sensitivity Analysis	Likely deterministic (range reporting)	Deterministic sensitivity analyses	One-way & scenario analyses	One-way sensitivity	Deterministic sensitivity analyses	Deterministic sensitivity analysis	Not stated	One-way sensitivity	One-way sensitivity & scenario analysis
Most expensive step	Targeted capture sequencing & bioinformatics analysis	Enrichment step (somatic & germline)	Next-Generation Sequencing interpretation and reporting	Library preparation & sequencing	Not stated	Library Preparation	ctDNA analysis step (library preparation, sequencing, & data analysis)	Library preparation and sequencing	DNA sequencing on NovaSeq
Least expensive step	Specimen collection, preparation, & quality assessment,	Biological validation (somatic); technical validation (germline)	Consent	Data storage	Not stated	Bioinformatics analysis	Blood sample collection step	Administrative support	Data storage
Main Cost Driver	Supplies and Reagents (FISH); Capital & Equipment (GEP & targeted capture sequencing)	Consumable	Consumables	Sequencing consumables & materials	Consumable	Consumable	Testing volume	Supply & reagent	Consumable
Equipment depreciation	Straight line amortization	5-year linear amortization	Not stated	Annuity-based depreciation; 5-year equipment lifespan; 3% interest rate	Annuity-based depreciation; equipment lifespan 5–10 years; interest rate ~ 4-4.5%	Straight-line depreciation over 5 years; 3% interest rate	Straight-line depreciation; 5-year lifespan; 4.2% interest rate	Amortisation over equipment lifespan; 1.5% annual cost of capital	Not stated

**Table 5:** Cost estimates (original and standardized)

	Original currency & year	Unit Cost	Original cost estimate (reported)^a^		Inflated, adjusted total costs (2024 USD)^a, b^	
Costa et al. (2016) [48]	Canadian dollars (2014)	Mean per-case	Targeted capture sequencing and bioinformatics: 1029.16		Targeted capture sequencing and bioinformatics: 1089.97	
			FISH: 596.60		FISH: 631.85	
			Digital GEP: 898.35		Digital GEP: 951.43	
Marino et al. (2018) [49]	Euro (2014)	Per person/patient	Somatic: 607.00 ± 207.00		Somatic: 945.43 ± 322.41	
			Germline: 550.00 ± 140.00		Germline: 856.65 ± 218.06	
Ryan et al. (2019) [50]	Pound sterling (2017)	Per case	Tumour triage with MSI and reflex MLH1 methylation testing followed by germline NGS: 42.01		Tumour triage with MSI and reflex MLH1 methylation testing followed by germline NGS: 76.91	
			Tumour triage with IHC and reflex MLH1 methylation testing of MLH1 protein-deficient cancers followed by NGS: 45.68		Tumour triage with IHC and reflex MLH1 methylation testing of MLH1 protein-deficient cancers followed by NGS: 83.63	
			Tumour triage with MSI followed by NGS: 78.95		Tumour triage with MSI followed by NGS: 144.55	
			NGS: 176.24		NGS:322.67	
Gordon et al. (2020) [51]	Australian dollars (2018)	Per person/patient	Targeted panels: 347.00-429.00		Targeted panels: 290.97-359.73	
			WES: 871.00-2788.00		WES: 730.35-2337.80	
			WGS: 2895.00-4830.00		WGS: 2427.52-4050.05	
Pasmans et al. (2021) [52]	Euro (2018)	Per person/patient	Sanger sequencing, three amplicons: 58.00		Sanger sequencing, three amplicons: 91.16	
			Paired tumour-normal WGS:2925.00		Paired tumour-normal WGS: 4597.48	
Bayle et al. (2021) [53]	Euro (2015; 2018)	Per sample & Per person	2015: 1921.00 per sample (i.e. cost of 3842.00 per person)		2015: 2956.51 per sample (i.e. cost of 5913.02 per person)	
			2018: 804.00 per sample (i.e. cost of 1608.00 per person)		2018: 1212.62 per sample (i.e. cost of 2425.24 per person)	
Kramer et al. (2022) [54]	Euro (2020)	Per sample	Digital droplet PCR: 168.00-1200.00		Digital droplet PCR: 251.44-1796.02	
			Real-time PCR 1: 643.00-1583.00		Real-time PCR 1: 962.37-2369.25	
			Real time PCR 2: 477.00-970.00		Real time PCR 2: 713.92 -1451.78	
			Mass spectrometry: 407.00-2223.00		Mass spectrometry: 609.15-3327.13	
			Commercial NGS: 1423.00-7100.00		Commercial NGS: 2129.78-10626.45	
			Custom NGS: 500.00-7713.00		Custom NGS: 748.34-11543.91	
Kumar et al. (2022) [55]	Canadian dollars (2019)	Per sample	DNA sample	Trusight Tumour 170 Kit: 1287.87	DNA sample	Trusight Tumour 170 Kit: 1290.17
				SeqCap EZ/KAPA Hyper Prep Plus Custom: 1227.93		SeqCap EZ/KAPA Hyper Prep Plus Custom: 1230.13
				Oncomine Focus: 1005.33		Oncomine Focus: 1007.13
				QIAseq Targeted DNA Custom Panel: 449.32		QIAseq Targeted DNA Custom Panel: 450.12
			RNA sample	Trusight Tumour 170 Kit: 1245.95	RNA sample	Trusight Tumour 170 Kit: 1248.18
				Oncomine Focus: 1001.75		Oncomine Focus: 1003.5
				QIASeq Targeted RNAscan Custom Panel: 586.70		QIASeq Targeted RNAscan Custom Panel: 587.75
Thangavelu et al. (2024) [56]	Euro (2023)	Per person/patient	2500 analyses: 3472		2500 analyses: 408.57	
			7500 analyses: 2671		7500 analyses: 314.31	

*FISH* Fluorescence in situ hybridization, *GEP* Digital gene expression profiling, *MSI* microsatellite instability, *IHC* immunohistochemistry, *NGS* next generation sequencing, *WES* Whole exome sequencing, *WGS* Whole genome sequencing, *PCR* Polymerase Chain Reaction;

^a^ Cost for type of genetic and/or genomic testing as listed in Table 2

^b^ Costs were converted to USD 2024, using the CCEMG-EPPI-Centre Cost Converter [23]

### Quality assessment

Most of the studies scored highly according to the CHEC checklist. All the included studies (*n* = 9, 100%) [48–56] demonstrated well-designed research questions for a clearly defined population. Besides, the studies also evaluated those relevant alternatives, adopted an appropriate perspective, as well as utilized a suitable economic study design that aligned with the stated objectives and conclusions. Additionally, all the studies (*n* = 9, 100%) [48–56] reported conflicts of interest and discussed the generalizability of their results to other settings and patient/client groups. However, not all studies required ethical approval, with only three studies explicitly obtaining it [48, 50, 51]. All the studies performed sensitivity analyses except two studies [48, 54] did not specify the type of sensitivity analysis conducted. The detailed results of the quality assessment for the included studies were available in Additional File 1 Table S7.

## Discussion

This systematic review evaluates the micro-costing methods used to estimate the costs of various laboratory-based genetic and genomic testing in oncology. Direct comparisons across all the nine included studies are challenging due to the differences in cost components and workflows. However, this study advances the prior reviews by identifying systematic gaps in cost data, clarifying challenges specific to cancer genetic and genomic testing, and emphasizing practical methodological elements to inform standardized micro-costing frameworks for HTA.

Methodologically, majority of the studies employed micro-costing analysis within observational comparative study designs [23–27, 30, 31]. Multi-centre data was the most common data collection approach [24, 26, 27, 29, 31]. Studies conducted in multi-centre settings may provide more generalizable findings but require higher operational costs compared to single-centre studies. However, conducting studies at a limited number of facilities poses challenges in translating results to other settings such as adjusting labour inputs to account for the differences in worker qualifications between the original and new settings [10]. All the studies were conducted from a healthcare provider perspective [23–31] which provided a better understanding of the financial burden on healthcare systems and informed resource allocation decisions.

Unlike many other health interventions, laboratory-based genetic and genomic testing in oncology is characterised by high analytical complexity, iterative interpretation processes, and rapidly evolving workflows [10]. Notably, this review focuses specifically on cancer-related genetic and genomic testing, distinguishing it from prior works such as Gonzalez et al. [15], which included rare diseases. Although no existing systematic review explicitly compares the cost of cancer testing versus rare disease testing, a gap identified by Mordaunt et al. [57]. Mordaunt et al. [57] recommended that future studies explore differences between constitutional, somatic, rare disease, and cancer genomic testing A pertinent example is Schwarze et al. [16], a micro-costing study of Illumina-based genome sequencing in a UK NHS laboratory, which reported separate estimates (£6841 per cancer case for matched tumour-germline versus £7050 per rare disease trio). However, it was excluded from our review due to its mixed population (cancer and rare diseases). Although the cost estimates were disaggregated, shared laboratory infrastructure, equipment allocation, and overheads may influence resource use in ways that are not fully separable for cancer-specific analysis. This decision prioritises methodological homogeneity and minimises potential confounding, thereby strengthening the validity of our findings for informing oncology policy and reimbursement decisions.

Cancer testing typically requires deeper sequencing coverage, tumour-normal comparisons, complex bioinformatic pipelines, and multidisciplinary interpretation to distinguish somatic, germline, and clinically actionable variants [58, 59]. All these features demand substantial resources especially consumable, skilled personnel and data analysis. These complexities are not readily captured using conventional micro-costing approaches developed for pharmaceutical or diagnostic imaging. The findings of this review demonstrate that current studies frequently underestimate these complexities, particularly failing to account for bioinformatics, data storage, and software maintenance. Significant methodological heterogeneity characterizes the identification, measurement and valuation of laboratory inputs, thereby limiting cross-study comparability. Direct observation was the predominant strategy applied for identification and measurement. This method is favoured for its objectivity in quantifying resource use and workforce time allocation, but it is resource-intensive and susceptible to the Hawthorne effect, often necessitating mitigation through longitudinal sampling or triangulation [60]. Conversely, while expert consensus and staff interviews provide first-hand operational knowledge, they carry an inherent risk of recall bias [61]. Valuation methods also varied widely. Market-based and list pricing (manufacturer, supplier, or catalogue prices) were the most common valuation methods. This valuation method is useful for facilitating wide comparisons, but it frequently fails to account for context-specific factors such as procurement discounts [62] or supply chain volatility [63]. Hence, mixed-method frameworks that integrate multiple data sources and costing techniques should be prioritized in micro-costing studies in cancer genetics or genomics. For example, combining bottom-up direct observation or time-and-motion studies (for precise measurement of labour-intensive steps like sequencing) with top-down allocation of indirect costs (e.g., from administrative records for equipment, data storage, and software) offers a balanced approach to accuracy and feasibility.

Comprehensive micro-costing studies were uncommon. Direct cost inputs such as consumables and reagents were consistently reported and emerged as dominant cost drivers [49–53, 55, 56]. In contrast, indirect costs were inconsistently defined and frequently underreported. Bioinformatics and data management which should be the core components of modern cancer genomics were often excluded or subsumed under poorly specified “overhead” categories. This pattern suggests a structural gap in how genomic laboratory work in reporting and considered in economic evaluations of the process. Failure to capture these resource-intensive components risks systematic underestimation of true delivery costs as cancer genomic testing becomes increasingly integrated into routine clinical care [53, 54, 64]. Inconsistency and lack of transparency in reporting all these methods remain critical barriers to reproducibility. Therefore, future micro-costing studies in cancer genetics or genomics should standardize the classification of inputs and explicitly distinguish laboratory consumables, personnel time, bioinformatics infrastructure, data storage, software licensing, quality assurance, and other indirect support components, rather than aggregating them under generic overhead categories. This would improve comparability, reproducibility, and policy relevance.

Findings also revealed that significant heterogeneity with weak justification of key assumptions applied in all the included studies [48–56]. Parameters such as equipment depreciation lifetimes, discount rates, and valuation sources were rarely justified or explored through sensitivity analyses. These findings indicate that the challenge is not merely lack of standardisation, but also the absence of oncology-specific methodological norms for micro-costing genetic and genomic tests. No single study comprehensively addressed all these elements. However, a minority of studies demonstrated stronger practice in specific domains, such as use of administrative data to value inputs [50, 51, 54] or partial inclusion of bioinformatics costs [53, 55, 56]. These studies provide useful reference points but also underscore the need for a more comprehensive and oncology-specific micro-costing framework.

Importantly, this review identifies gaps in the literature where genetic or genomic testing is recommended in clinical practice but robust micro-costing data are absent or sparse. Examples include reflex tumour sequencing pathways, paired tumour–normal testing, repeat testing at disease progression, and integrated liquid biopsy strategies for treatment monitoring. These applications remain underrepresented in the economic evidence which limits the ability of policymakers to assess affordability, scalability, and value for money despite their growing clinical relevance. Targeted micro-costing studies in these areas would be particularly informative, especially in settings where resource constraints necessitate careful prioritisation.

### Strengths and limitations

This systematic review provided important insight into the methodology and detailed cost analysis of resources used for laboratory genetic and genomic testing for cancer. Beyond summarizing the latest findings, this study assessed study quality and methodology, while identifying research gaps for future exploration. The scope here is broader than Gonzalez et al., who limited their review to exome and genomic sequencing [15]. Additionally, this review also discussed the trade-offs between estimate accuracy and data availability for each cost component or resource. This highlights how mixed methods such as the integration of observational and administrative or records data can help to improve cost estimations. Nevertheless, the limitations of this review still exist. Firstly, the restriction to English-language articles might lead to the exclusion of valuable publications in other languages which might potentially be limiting the comprehensiveness of the review. Secondly, this review might have overlooked other relevant studies due to a lack of standardization in micro-costing terminology. Thirdly, this review was restricted to oncology unlike Gonzalez et al. [15], which included both cancer and rare diseases. Only nine studies met the eligibility criteria as most published economic studies of genetic and genomic testing in oncology did not provide detailed laboratory-level resource breakdowns. Our study findings are still consistent with Gonzalez et al. [15], who identified only seven eligible studies despite broader inclusion criteria. The limited number of standalone cancer-focused micro-costing studies highlights the need for methodological standardisation and oncology-specific micro-costing studies to inform reimbursement, policy development, and service planning. Fourthly, the challenge of comparing costs across health systems with different financing and procurement structures still exists even after PPP adjustments were applied to improve cross-country comparability. Finally, the rapidly evolving technologies mean that older studies may have reported outdated prices, though their methodological insights remain valuable.

### Suggestions for future research

The findings suggest the calls for standardisation must extend beyond general reporting transparency to specify minimum methodological requirements tailored to cancer genetic and genomic testing. Part of the guideline should include estimating economic costs in a manner that allows for meaningful comparison between studies. Future research should adopt oncology-specific methodological norms in the micro-costing study of genetic or genomic testing, including:

- Explicitly report the complete laboratory workflow, from pre-analytical processing through sequencing, bioinformatics, variant interpretation, and reporting, rather than focusing solely on assay-level inputs.
- Clearly describe the testing pathway, including any reflex or repeat analyses.
- Transparently report the direct and indirect costs.
- Apply mixed-method frameworks (e.g., combining observational and administrative data) for resource identification, measurement, and valuation.
- Clearly define and disaggregate indirect costs, particularly for bioinformatics infrastructure, software licenses, data storage, training, facility expenses, and failure rates related to oncology testing.
- Justify assumptions for equipment depreciation and discount rates.
- Explicitly report excluded cost components.
- Standardize the unit of analysis around testing pathways (e.g., cost per sample, per patient) and distinguish between tumour-only and tumour-normal sequencing.
- Consider testing volume and batching effects.

## Conclusion

This systematic review highlights significant variability in the methodological approaches and cost estimates used in micro-costing studies of genetic and genomic testing for cancer. Although micro-costing is considered the gold standard for cost assessment in economic evaluations, the differences in sources of data as well as measurement and valuation techniques employed, and the cost components used within and between studies impede comparability and generalizability. WES and WGS remain the most expensive genetic and/or genomic testing technologies. In all of the studies reviewed, the key cost drivers were consumables and reagents whereas the most expensive steps were library preparation and sequencing. Policymakers need to consider cost-driving factors, volume-based pricing approaches, and transparent reporting standards in order to promote and implement effective genetic and genomic testing measures. Economic evaluations and decision-making in the context of constrained resources will be better supported if future work adopted standardized frameworks and defined cost components more clearly.

## Supplementary Information

Supplementary Material 1.

## Data Availability

All data generated or analysed during this study are included in this published article and its supplementary information.

## Abbreviations

BMA: British Medical Association
BIA: Budget Impact Analysis
CC: Campbell Collaboration
CEA: Cost-Effectiveness Analysis
CHEC: Consensus on Health Economic Criteria
CINAHL: Cumulative Index of Nursing and Allied Health Literature
ctDNA: circulating tumour DNA
GDP: Gross Domestic Product
HTA: Health Technology Assessment
IHC: Immunohistochemistry
LMICs: Low- And Middle-Income Countries
MeSH: Medical Subject Headings
NCDs: Noncommunicable Diseases
NGS: Next-Generation Sequencing
NHS: National Health Service
PPP: purchasing power parity
PRISMA: Preferred Reporting Items for Systematic Reviews and Meta-Analyses
PROSPERO: International Prospective Register of Systematic Reviews
SOP: Standard Operating Procedure
WES: Whole Exome Sequencing
WGS: Whole-Genome Sequencing
