# Supplementary material for: Micro-costing of genetic and genomic testing in oncology: a systematic review of laboratory resources and costs
Source: Health Econ Rev. 2026 Mar 5;16:57. doi: 10.1186/s13561-026-00752-w (PMC13147868; doi:10.1186/s13561-026-00752-w)
Supplement: Supplementary file 1 — Supplementary Material 1. [file 13561_2026_752_MOESM1_ESM.docx]

**Title**

Micro-Costing of Genetic and Genomic Testing in Oncology: A Systematic Review of Laboratory Resources and Costs

**Journal Name**

Health Economic Review

**Authors Information**

1. Sook Pin Goh **(first author)**

Discipline of Social and Administrative Pharmacy, School of Pharmaceutical Sciences, Universiti Sains Malaysia, Penang, Malaysia

gohpin1219@gmail.com

ORCID: 0009-0008-5173-1686

1. Jue Ern Chan

[jueern@gmail.com](mailto:jueern@gmail.com)

1. Siew Chin Ong **(corresponding author)**

Discipline of Social and Administrative Pharmacy

School of Pharmaceutical Sciences

Universiti Sains Malaysia

Penang, Malaysia

siewchinong@usm.my

ORCID: 0000-0002-9750-9588

Supplementary Information

**Table S1: Search terms**

| **Category** | **Search Term** |
| --- | --- |
| **Cost** | Cost  Economic  Micro-costing  Bottom-up costing  Top-down costing  Activity-based costing |
| **Genetic Testing** | Genetic Testing  Genomic Testing  Genetic Sequencing  Germline Testing  Germline Sequencing  Whole Genome Sequencing  Whole Exome Sequencing  Targeted gene panels |
| **Cancer** | Neoplasm  Tumor  Tumour  Cancer |

**Table S2: Search Results of PUBMED (dated: 2/11/2024)**

| **Set Number** | **Search Term for Pubmed** | **Results** |
| --- | --- | --- |
| #1 | "cost*"[All Fields] OR "economic*"[All Fields] | 1,772,287 |
| #2 | "bottom up"[Title/Abstract] OR "top down"[Title/Abstract] OR "activity based costing"[Title/Abstract] OR "abc method"[Title/Abstract] OR "activity based cost*"[Title/Abstract] OR "microcosting"[Title/Abstract] | 37,370 |
| #3 | "time and motion studies"[MeSH Terms] OR "time study"[Title/Abstract] OR "time motion"[Title/Abstract] OR "time-and-motion"[Title/Abstract] | 8,365 |
| #4 | #2 OR #3 | 45,651 |
| #5 | #1 AND #4 | [6,680](https://pubmed.ncbi.nlm.nih.gov/?term=%28%28cost%2A%29+OR+%28economic%2A%29%29+AND+%28%28%28%28%28%28%28bottom+up%5BTitle%2FAbstract%5D%29+OR+%28top+down%5BTitle%2FAbstract%5D%29%29+OR+%28activity-based+costing%5BTitle%2FAbstract%5D%29%29+OR+%28ABC+method%5BTitle%2FAbstract%5D%29%29+OR+%28activity+based+cost%2A%5BTitle%2FAbstract%5D%29%29+OR+%28microcosting%5BTitle%2FAbstract%5D%29%29+OR+%28%28%28%28time+and+motion+studies%5BMeSH+Terms%5D%29+OR+%28time+study%5BTitle%2FAbstract%5D%29%29+OR+%28time+motion%5BTitle%2FAbstract%5D%29%29+OR+%28time-and-motion%5BTitle%2FAbstract%5D%29%29%29&sort=) |
| #6 | "genetic testing"[MeSH Terms] OR "genetic test*"[Title/Abstract] OR "genetic screen*"[Title/Abstract] OR "genomic test*"[Title/Abstract] OR "genomic screen*"[Title/Abstract] OR "hereditary test*"[Title/Abstract] OR "dna test*"[Title/Abstract] OR "dna screen*"[Title/Abstract] OR "whole genome sequencing"[MeSH Terms] OR "whole genome sequence*"[Title/Abstract] OR "WGS"[Title/Abstract] OR "targeted gene panel*"[Title/Abstract] OR "targeted sequence*"[Title/Abstract] OR "multigene panel*"[Title/Abstract] OR "Molecular Diagnostic Techniques"[MeSH Terms] OR "molecular diagnostic test*"[Title/Abstract] OR "molecular diagnosis"[Title/Abstract] OR (("germline"[All Fields] OR "germlines"[All Fields]) AND "research design"[MeSH Terms]) OR "germline test*"[Title/Abstract] OR "germline sequence*"[Title/Abstract] OR "exome sequencing"[MeSH Terms] OR "whole exome sequence*"[Title/Abstract] OR "WES"[Title/Abstract] | 172,192 |
| #7 | "neoplasms"[MeSH Terms] OR "neoplasm*"[Title/Abstract] OR "tumor*"[Title/Abstract] OR "tumour*"[Title/Abstract] OR "cancer*"[Title/Abstract] | 5,217,897 |
| #8 | #5 AND #6 AND #7 | 32 |
| #9 | #8 AND Filters: English, from 2005/1/1 - 2024/10/31 | 30 |

**Table S3: Search Results of SCOPUS (dated: 3/11/2024)**

| **Set Number** | **Search Term for Scopus** | **Results** |
| --- | --- | --- |
| #1 | ( TITLE-ABS-KEY ( cost* ) OR TITLE-ABS-KEY ( economic* ) ) | 6,413,447 |
| #2 | ( TITLE-ABS-KEY ( microcosting ) ) OR ( TITLE-ABS-KEY ( {top down} ) ) OR ( TITLE-ABS-KEY ( {top-down} ) ) OR ( TITLE-ABS-KEY ( {bottom up} ) ) OR ( TITLE-ABS-KEY ( {bottom-up} ) ) OR ( TITLE-ABS-KEY ( {activity-based costing} ) ) OR ( TITLE-ABS-KEY ( {activity based costing} ) ) OR ( TITLE-ABS-KEY ( {ABC method} ) ) | 124,999 |
| #3 | ( TITLE-ABS-KEY ( {time study} ) OR TITLE-ABS-KEY ( {time motion} ) OR TITLE-ABS-KEY ( time-and-motion ) OR TITLE-ABS-KEY ( {time and motion} ) ) | 15,495 |
| #4 | #2 OR #3 | 140,422 |
| #5 | #1 AND #4 | 22,757 |
| #6 | ( TITLE-ABS-KEY ( {genetic test*} ) ) OR ( TITLE-ABS-KEY ( {genetic screen*} ) ) OR ( TITLE-ABS-KEY ( {genomic test*} ) ) OR ( TITLE-ABS-KEY ( {genomic screen*} ) ) OR ( TITLE-ABS-KEY ( {hereditary test*} ) ) OR ( TITLE-ABS-KEY ( {DNA test*} ) ) OR ( TITLE-ABS-KEY ( {DNA screen*} ) ) OR ( TITLE-ABS-KEY ( {whole genome sequence*} ) ) OR ( TITLE-ABS-KEY ( wgs ) ) OR ( TITLE-ABS-KEY ( {whole exome sequence*} ) ) OR ( TITLE-ABS-KEY ( wes ) ) OR ( TITLE-ABS-KEY ( {targeted gene panel*} ) ) OR ( TITLE-ABS-KEY ( {targeted sequence*} ) ) OR ( TITLE-ABS-KEY ( {multigene panel*} ) ) OR ( TITLE-ABS-KEY ( {Molecular Diagnostic Techniques} ) ) OR ( TITLE-ABS-KEY ( {molecular diagnostic test*} ) ) OR ( TITLE-ABS-KEY ( {molecular diagnosis} ) ) OR ( TITLE-ABS-KEY ( {germline test*} ) ) OR ( TITLE-ABS-KEY ( {germline sequence*} ) ) | 69,391 |
| #7 | (TITLE-ABS-KEY(neoplasm*)) OR (TITLE-ABS-KEY(tumor*)) OR (TITLE-ABS-KEY(tumour*)) OR (TITLE-ABS-KEY(cancer*)) | 6,343,047 |
| #8 | #5 AND #6 AND #7 | 9 |
| #9 | #8 AND PUBYEAR > 2004 AND PUBYEAR < 2025 AND ( LIMIT-TO ( LANGUAGE , "English" ) | 8 |

**Table S4: Search Results of Web of Science (Dated: 3/11/2024)**

| **Set Number** | **Search Term for Pubmed** | **Results** |
| --- | --- | --- |
| #1 | TS=(cost*) OR TS=(economic*)) | 6,634,103 |
| #2 | (TS=(microcosting) OR TS=(top-down) OR TS=(bottom-up) OR TS=("activity-based costing") OR TS=("ABC method")) | 143,241 |
| #3 | (((TS=("time study")) OR TS=("time motion")) OR TS=(time-and-motion )) OR TS=("time and motion") | 17,246 |
| #4 | #2 OR #3 | 160,382 |
| #5 | #1 AND #4 | 28,429 |
| #6 | (((((((((((((((((((TS=("genetic test*" )) OR TS=("genetic screen*")) OR TS=("genomic test*")) OR TS=("genomic screen*")) OR TS=("hereditary test*")) OR TS=("DNA test*")) OR TS=("DNA screen*")) OR TS=("whole genome sequence*")) OR TS=(WGS)) OR TS=("whole exome sequence*")) OR TS=(WES)) OR TS=("targeted gene panel*")) OR TS=("targeted sequence*")) OR TS=("multigene panel*")) OR TS=("Molecular Diagnostic Techniques")) OR TS=("molecular diagnostic test*")) OR TS=("molecular diagnosis")) OR TS=("germline test*")) OR TS=("germline sequence*")) | 214,148 |
| #7 | (((TS=(neoplasm*)) OR TS=(tumo$r*)) OR TS=(cancer*)) | 7,785,034 |
| #8 | #5 AND #6 AND #7 | 24 |
| #9 | #8 and Timespan: 2005-01-01 to 2024-10-31 (Publication Date); Language: English | 20 |

**Table S5: Search Results of CINAHL (dated: 3/11/2024)**

| **Set Number** | **Search Term for Pubmed** | **Results** |
| --- | --- | --- |
| #1 | TI cost* OR AB cost* OR TI economic* OR AB economic* | 1,593,860 |
| #2 | TI microcosting OR AB microcosting OR TI "top down" OR AB "top down" OR TI "bottom up" OR AB "bottom up" OR TI "activity based costing" OR AB "activity based costing" | 44,279 |
| #3 | TI "ABC method" OR AB "ABC method" | 961 |
| #4 | TI "time study" OR AB "time study" OR TI "time motion" OR AB "time motion" OR TI "time-and-motion" OR AB "time-and-motion" OR TI ( "time and motion" ) OR AB ( "time and motion" ) | 4,189 |
| #5 | #2 OR #3 OR #4 | 49,347 |
| #6 | #1 AND #5 | 6,610 |
| #7 | TI "genetic test*" OR AB "genetic test*" OR TI "genetic screen*" OR AB "genetic screen*" OR TI "genomic test*" OR AB "genomic test*" OR TI "genomic screen*" OR AB "genomic screen*" OR TI "hereditary test*" OR AB "hereditary test*" OR TX "DNA test*" OR AB "DNA test*" | 71,051 |
| #8 | TI "DNA screen*" OR AB "DNA screen*" OR TI "whole genome sequence*" OR AB "whole genome sequence*" OR TI WGS OR AB WGS OR TI "whole exome sequence*" OR AB "whole exome sequence*" OR TI WES OR AB WES OR TX "targeted gene panel" OR AB "targeted gene panel" | 30,780 |
| #9 | TI "targeted sequence*" OR AB "targeted sequence*" OR TI "multigene panel" OR AB "multigene panel" OR TI "Molecular Diagnostic Techniques" OR AB "Molecular Diagnostic Techniques" OR TI "molecular diagnostic test*" OR AB "molecular diagnostic test*" OR TI "molecular diagnosis" OR AB "molecular diagnosis" | 14,676 |
| #10 | TI "germline test*" OR AB "germline test*" OR TI "germline sequence*" OR AB "germline sequence*" | 1,193 |
| #11 | #7 OR #8 OR #9 OR #10 | 114,289 |
| #12 | TI neoplasm OR AB neoplasm OR TI tumo#r OR AB tumo#r OR TI cancer OR AB cancer | 3,665,491 |
| #13 | #6 AND #11 AND #12 | 14 |
| #14 | #13   \|  \| Limiters - Publication Date: 20050101-20241031 \| \| --- \| --- \| | 13 |

**Table S6: Rationale for excluded studies in systematic review**

| **Study** | **Reason** |
| --- | --- |
| Johnson Y, Goldberg P, Moodley J, Algar U, Thomson S, Sinanovic E, Ramesar R. A comparative cost analysis of two screening strategies for colorectal cancer in Lynch Syndrome in a South African tertiary hospital. Cancer Causes & Control. 2023 Feb;34(2):161-9. | Different study types/ interventions  - Included other screening strategies (Colonoscopy) |
| Ginindza TG, Sartorius B, Dlamini X, Östensson E. Cost analysis of Human Papillomavirus-related cervical diseases and genital warts in Swaziland. PloS one. 2017 May 22;12(5):e0177762. | No genetic testing |
| Levin CE, Sellors J, Shi JF, Ma L, Qiao YL, Ortendahl J, O'Shea MK, Goldie SJ. Cost‐effectiveness analysis of cervical cancer prevention based on a rapid human papillomavirus screening test in a high‐risk region of China. International journal of cancer. 2010 Sep 15;127(6):1404-11. | No detailed breakdown of laboratory resource costs |
| Dharampal N, Smith K, Harvey A, Paschke R, Rudmik L, Chandarana S. Cost-effectiveness analysis of molecular testing for cytologically indeterminate thyroid nodules. Journal of Otolaryngology-Head & Neck Surgery. 2022 Jan;51(1):46. | No detailed breakdown of laboratory resource costs |
| MacKay C, Turner B, Clarke S, Wallace T, Rigby MH. Cost-Effectiveness Analysis of Molecular Testing for Indeterminate Thyroid Nodules in Nova Scotia. Journal of Otolaryngology-Head & Neck Surgery. 2024 Oct;53:19160216241291806. | No detailed breakdown of laboratory resource costs |
| Campos NG, Tsu V, Jeronimo J, Njama-Meya D, Mvundura M, Kim JJ. Cost-effectiveness of an HPV self-collection campaign in Uganda: comparing models for delivery of cervical cancer screening in a low-income setting. Health policy and planning. 2017 Sep 1;32(7):956-68. | No detailed breakdown of laboratory resource costs |
| Ramdzan AR, Manaf MR, Aizuddin AN, Latiff ZA, Teik KW, Ch'ng GS, Ganasegeran K, Aljunid SM. Cost-effectiveness of colorectal cancer genetic testing. International Journal of Environmental Research and Public Health. 2021 Aug 6;18(16):8330. | No detailed breakdown of laboratory resource costs |
| Campos NG, Mvundura M, Jeronimo J, Holme F, Vodicka E, Kim JJ. Cost-effectiveness of HPV-based cervical cancer screening in the public health system in Nicaragua. BMJ open. 2017 Jun 1;7(6):e015048. | No detailed breakdown of laboratory resource costs |
| Snowsill TM, Ryan NA, Crosbie EJ. Cost-effectiveness of the Manchester approach to identifying Lynch syndrome in women with endometrial cancer. Journal of Clinical Medicine. 2020 Jun 1;9(6):1664. | No detailed breakdown of laboratory resource costs |
| Lince-Deroche N, Phiri J, Michelow P, Smith JS, Firnhaber C. Costs and cost effectiveness of three approaches for cervical cancer screening among HIV-positive women in Johannesburg, South Africa. PLoS One. 2015 Nov 16;10(11):e0141969. | No detailed breakdown of laboratory resource costs |
| Hariprasad R, Bagepally BS, Kumar S, Pradhan S, Gurung D, Tamang H, Sharma A, Bhatnagar T. Cost-utility analysis of primary HPV testing through home-based self-sampling in comparison to visual inspection using acetic acid for cervical cancer screening in East district, Sikkim, India, 2023. Plos one. 2024 Aug 13;19(8):e0300556. | No detailed breakdown of laboratory resource costs |
| Vu M, Degeling K, Ryland GL, Hofmann O, Ng AP, Westerman D, IJzerman MJ. Economic Impact of Whole Genome Sequencing and Whole Transcriptome Sequencing Versus Routine Diagnostic Molecular Testing to Stratify Patients with B-Cell Acute Lymphoblastic Leukemia. The Journal of Molecular Diagnostics. 2024 Aug 1;26(8):673-84. | Different study types/ interventions:  Cost-effectiveness analysis; micro-costing not the primary study objective |
| Sabatini LM, Mathews C, Ptak D, Doshi S, Tynan K, Hegde MR, Burke TL, Bossler AD. Genomic sequencing procedure microcosting analysis and health economic cost-impact analysis: a report of the Association for Molecular Pathology. The Journal of Molecular Diagnostics. 2016 May 1;18(3):319-28. | Different study types/ interventions  -Review paper |
| Patel YP, Husereau D, Leighl NB, Melosky B, Nam J. Health and budget impact of liquid-biopsy-based comprehensive genomic profile (CGP) testing in tissue-limited advanced non-small cell lung cancer (aNSCLC) patients. Current Oncology. 2021 Dec 11;28(6):5278-94. | No detailed breakdown of laboratory resource costs |
| Accetta G, Biggeri A, Carreras G, Lippi G, Carozzi FM, Confortini M, Zappa M, Paci E. Is human papillomavirus screening preferable to current policies in vaccinated and unvaccinated women? A cost-effectiveness analysis. Journal of medical screening. 2010 Dec;17(4):181-9. | No detailed breakdown of laboratory resource costs |
| Van Amerongen RA, Retèl VP, Coupé VM, Nederlof PM, Vogel MJ, Van Harten WH. Next-generation sequencing in NSCLC and melanoma patients: a cost and budget impact analysis. Ecancermedicalscience. 2016;10. | Different study types/ interventions  - Include budget impact analysis (BIA) study |
| Blons H, Rouleau E, Charrier N, Chatellier G, Côté JF, Pages JC, de Fraipont F, Boyer JC, Merlio JP, Morel A, Gorisse MC. Performance and cost efficiency of KRAS mutation testing for metastatic colorectal cancer in routine diagnosis: the MOKAECM study, a nationwide experience. PLoS One. 2013 Jul 25;8(7):e68945. | No detailed breakdown of laboratory resource costs |
| Darlington M, Sujobert P, Kosmider O, Luque Paz D, Kaltenbach S, Figeac M, Hayette S, Mezaour N, Coquerelle S, Alary AS, Bidet A. Targeted High‐throughput Sequencing for Hematological Malignancies: A GBMHM Survey of Practice and Cost Evaluation in France. HemaSphere. 2023 Sep;7(9):e943. | No detailed breakdown of laboratory resource costs |
| \| Schwarze K, Buchanan J, Fermont JM, Dreau H, Tilley MW, Taylor JM, Antoniou P, Knight SJ, Camps C, Pentony MM, Kvikstad EM. The complete costs of genome sequencing: a microcosting study in cancer and rare diseases from a single center in the United Kingdom. Genetics in Medicine. 2020 Jan 1;22(1):85-94. \| \| --- \| | Mixed cancer and non-cancer population; review restricted to cancer-only studies. |
| Pagès A, Foulon S, Zou Z, Lacroix L, Lemare F, De Baère T, Massard C, Soria JC, Bonastre J. The cost of molecular-guided therapy in oncology: a prospective cost study alongside the MOSCATO trial. Genetics in Medicine. 2017 Jun 1;19(6):683-90. | No detailed breakdown of laboratory resource costs |
| Tan RY, Met-Domestici M, Zhou K, Guzman AB, Lim ST, Soo KC, Feeley TW, Ngeow J. Using quality improvement methods and time-driven activity-based costing to improve value-based cancer care delivery at a cancer genetics clinic. Journal of oncology practice. 2016 Mar;12(3):e320-31. | No detailed breakdown of laboratory resource costs |
| Griffith GL, Tudor-Edwards R, Gray J, Butler R, Wilkinson C, Turner J, France B, Bennett P. A micro costing of NHS cancer genetic services. British journal of cancer. 2005 Jan;92(1):60-71. | Different study types/ interventions  - included genetic counselling |
| \| Pisapia P, Pepe F, Baggi A, Barberis M, Galvano A, Gristina V, Mastrilli F, Novello S, Pagni F, Pasini S, Perrone G. Next generation diagnostic algorithm in non-small cell lung cancer predictive molecular pathology: the KWAY Italian multicenter cost evaluation study. Critical Reviews in Oncology/hematology. 2022 Jan 1;169:103525. \| \| --- \| | No detailed breakdown of laboratory resource costs |
| Plöthner M, Frank M, von der Schulenburg JM. Cost analysis of whole genome sequencing in German clinical practice. The European Journal of Health Economics. 2017 Jun;18:623-33. | Different study types/ interventions  - does not explicitly focus on cancer-related genetic testing |

**Table S7 Quality assessment (Adapted and modified from CHEC Checklist)**

|  | **Costa et al. (2016)** | **Marino et al. (2018)** | **Ryan et al. (2019)** | **Gordon et al. (2020)** | **Pasmans et al. (2021)** | **Bayle et al. (2021)** | **Kramer et al. (2022)** | **Kumar et al. (2022)** | **Thangavelu et al. (2024)** |
| --- | --- | --- | --- | --- | --- | --- | --- | --- | --- |
| 1. Is the study population clearly defined? | Yes | Yes | Yes | Yes | Yes | Yes | Yes | Yes | Yes |
| 2. Are the competing alternatives clearly described? | Yes | Yes | Yes | Yes | Yes | Yes | Yes | Yes | Yes |
| 3. Is a well-designed research question posed in an answerable form? | Yes | Yes | Yes | Yes | Yes | Yes | Yes | Yes | Yes |
| 4. Is the economic study design appropriate to the stated objective? | Yes | Yes | Yes | Yes | Yes | Yes | Yes | Yes | Yes |
| 5. Is the actual perspective chosen appropriate? | Yes | Yes | Yes | Yes | Yes | Yes | Yes | Yes | Yes |
| 6. Sensitivity analysis performed | Yes (Not clearly stated type of sensitivity analysis) | Yes | Yes | Yes | Yes | Yes | Yes (Not clearly stated type of sensitivity analysis) | Yes | Yes |
| 7. Do the conclusions follow from the data reported? | Yes | Yes | Yes | Yes | Yes | Yes | Yes | Yes | Yes |
| 8. Does the study discuss the generalizability of the results to other settings and patient/client groups? | Yes | Yes | Yes | Yes | Yes | Yes | Yes | Yes | Yes |
| 9. Do the authors reports any conflict of interest? | Yes | Yes | Yes | Yes | Yes | Yes | Yes | Yes | Yes |
| 10. Was ethical approval obtained for the study? | Yes | Not stated/Not Clear | Yes | Yes | Not Applicable | Not Applicable | Not Applicable | Not Applicable | Not stated/Not Clear |
